# Evaluation of the Effects of Coronavirus Vaccination Status on Inpatient Demographics and Clinical and Laboratory Data

**DOI:** 10.7759/cureus.47794

**Published:** 2023-10-27

**Authors:** Ayten Girgin, Fatih Ileri, Senem Kaya, Nizameddin Koca

**Affiliations:** 1 Department of Internal Medicine, Bursa City Hospital, Bursa, TUR

**Keywords:** pandemic, outcomes, covid-19, vaccination status, breakthrough infection

## Abstract

Background

The coronavirus disease 2019 (COVID-19) pandemic has been largely controlled by vaccines. However, a notable increase in COVID-19 infections has been observed among vaccinated individuals. The protection conferred by vaccination remains a topic of ongoing discussion and research. Our study aims to assess the impact of vaccination status on the demographics, clinical presentations, and laboratory characteristics of patients who were admitted to the hospital and subsequently hospitalized for further evaluation and treatment.

Methods

We examined hospitalized COVID-19 patients in terms of demographics, immunization status, clinical and laboratory findings, and outcomes over a seven-month period during which the delta variant was prevalent. Patients were categorized into three groups based on their vaccination status: unvaccinated (n=1,321, 53.3%), partially vaccinated (n=214, 8.6%), and fully vaccinated (n=944, 38.1%). Data from these patients were compared across groups.

Results

The study included 2,479 polymerase chain reaction (PCR)-confirmed hospitalized COVID-19 patients. The median ages (range) for the unvaccinated, partially vaccinated, and fully vaccinated patients who required hospitalization due to COVID-19 infection were 51 (18-98), 61 (21-91), and 71 (23-99), respectively (p<0.001). White blood cell count, neutrophils, monocytes, platelet count, and inflammatory markers such as erythrocyte sedimentation rate, C-reactive protein, procalcitonin, and IL-6, as well as fibrinogen and troponin T levels, were observed to be higher in the fully vaccinated patients compared to the unvaccinated and partially vaccinated patients. Clinical follow-ups showed that the intensive care unit (ICU) admission rates, length of hospital stay, and mortality rates were also higher in the fully vaccinated group compared to the other groups.

Conclusion

Our findings indicate that full vaccination significantly reduces hospitalization rates in younger individuals with average risk. However, patients with high-risk factors, such as advanced age and multiple comorbidities, exhibited higher hospitalization rates, increased need for intensive care, longer hospital stays, elevated inflammatory markers, and higher mortality even when fully vaccinated. It is crucial for elderly patients to receive thorough evaluations during emergency visits and to be provided with early treatment to reduce potential morbidity and mortality.

## Introduction

Severe acute respiratory syndrome coronavirus 2 (SARS-CoV-2), from the family *Coronaviridae* spp., emerged at the end of 2019, spread rapidly worldwide, and was declared a pandemic by the World Health Organization in March 2020 [1,2]. Its clinical manifestations range from asymptomatic carriage and upper respiratory tract infection to moderate-to-severe pneumonia, multi-organ failure, and death [3,4]. By July 2022, over 575 million cases had been reported globally, resulting in more than six million deaths. Given the absence of effective antiviral treatments, vaccination emerges as the primary and most effective method to curb the spread of the coronavirus disease 2019 (COVID-19) pandemic.

Until July 2022, more than 15 million cases were reported in Turkey, and more than 99,000 deaths were encountered [5]. The Ministry of Health of Turkey started to use CoronaVac™ (Sinovac Biotech, Beijing, China) in January 2021 and Pfizer-BioNTech™ (BioNTech, Mainz, Germany) in April 2021 in the community vaccination program [6]. CoronaVac™ is a purified inactivated virus, and the primary vaccination regimen is two doses with an interval of two to four weeks [7]. In our country, a double-blind, randomized, phase 3 study involving 10,214 participants was conducted in 2021. The efficacy of the vaccine was found to be 83.5% [8]. The Pfizer-BioNTech™ vaccine is the first infectious disease vaccine developed using messenger RNA (mRNA) technology [9]. An efficacy of 95% has been demonstrated with two doses administered 21 days apart in individuals aged 16 and older [10]. Under the COVID-19 Vaccine National Implementation Strategy, the first groups to be vaccinated were healthcare workers, residents of nursing homes, and patients over the age of 65, who constituted the highest-risk group [11]. With the active vaccination strategy, as of July 2022, 85% of the population was vaccinated with at least two doses in Turkey [12].

Studies have indicated that the high efficacy of COVID-19 vaccines diminishes over time, with breakthrough infections particularly reported among elderly patients [13-16]. In a previous study, the incidence of the disease among elderly individuals who are fully vaccinated was 0.1% [17]. Analyzing this subset of patients, who exhibit a high mortality rate, will aid in refining our approach towards their care. The current study aims to evaluate the effects of vaccination status on inpatient demographics and clinical and laboratory characteristics of patients who were admitted to the hospital and decided to be hospitalized for follow-up and treatment after preliminary evaluation for COVID-19 infection confirmed by reverse transcription polymerase chain reaction (RT-PCR).

## Materials and methods

Study design and participants

A total of 2,479 COVID-19 patients, who tested positive via RT-PCR tests from nasopharyngeal swabs and were admitted to the tertiary care emergency department with COVID-19-related complaints between May 1 and November 30, 2021, were included in the study. The study excluded asymptomatic carriers who presented with non-COVID-related complaints, patients who tested positive for RT-PCR during pre-surgical evaluations, individuals under the age of 18, and pregnant patients diagnosed with COVID-19. Following the approval from the Bursa City Hospital Clinical Research Ethics Committee (Approval No: 2022-7/5), data on demographics (age, gender, and vaccination status), clinical and laboratory parameters at admission, intensive care unit (ICU) requirements, and length of stay data were obtained over the electronic medical records (EMR). Due to the retrospective nature of the study, informed consent has been waived.

In our country's coronavirus vaccination program, two vaccines were utilized: the mRNA-based Pfizer-BioNTech™ (BNT162b2) and the inactivated vaccine CoronaVac™. Both were administered in a two-dose regimen with a 28-day interval between doses. For the purpose of this study, patients were categorized into three groups: (1) unvaccinated (NoVac), (2) partially vaccinated (PV) (those who received a single dose of either Pfizer-BioNTech™ or CoronaVac™), and (3) fully vaccinated (FV) (those who received two doses of either Pfizer-BioNTech™ or CoronaVac™, administered 28 days apart).

Length of stay in inpatient and ICU hospitalization were recorded. The primary endpoint in clinical follow-up is determined as either discharge or death. Hospitalization and ICU decisions of the COVID-19 patients were made according to the algorithm in the guideline created and regularly updated by the Coronavirus Scientific Advisory Board of the Ministry of Health of Turkey [12]. Criteria for admission to intensive care according to this algorithm are as follows: (i) patients with dyspnea and respiratory distress (respiratory rate ≥30/min, PaO2/FiO2 <300, SpO2 <90%, or PaO2 <70 mmHg despite 5 L/min oxygen therapy); (ii) hypotension (systolic blood pressure (SBP) <90 mmHg and more than 40 mmHg drop from the usual SBP and mean arterial pressure < 65 mmHg); (iii) tachycardia >100/min; (iv) acute kidney injury; (v) impaired liver function tests; (vi) confusion; (vii) acute bleeding diathesis; (viii) patients with immunosuppression; (ix) troponin elevation and arrhythmia; and (x) patients with lactate >2 mmol and impaired circulation.

Statistical analysis

Our study retrospectively analyzed the patients' demographic, clinical, and laboratory data via EMR. The Shapiro-Wilk and Kolmogorov-Smirnov tests examined conformity to a normal distribution of continuous variables affecting the relationship between COVID-19 and vaccines. Continuous variables were expressed as median (minimum: maximum) and mean±standard deviation. Categorical variables were expressed as n(%). The independent sample t test, one-way analysis of variance (ANOVA), Mann-Whitney U test, and Kruskal-Wallis H test were used for intergroup comparisons of continuous variables. The Pearson chi-squared, Yates chi-squared, and Fisher-Freeman-Halton tests were used for intergroup comparisons of categorical variables. The data obtained in the study were evaluated in the IBM SPSS Statistics for Windows, Version 26.0 (Released 2019; IBM Corp., Armonk, New York, United States). Type 1 error level was accepted as 5% in statistical analysis.

## Results

This retrospective analysis was conducted using the EMR of 2,479 hospitalized patients (male/female: 1,279/1,200) admitted to our tertiary care center between May 1 and November 30, 2021. The median age of the study population was 59, ranging from 18 to 99 years. Patients were categorized based on their vaccination status in three groups: NoVac (n=1321, 53.3%), PV (n=214, 8.6%), and FV (n=944, 38.1%). Of the FV patients, 830 (87.90%) were vaccinated with CoronaVac™, 87 (9.20%) with Pfizer-BioNTech™, and 27 (2.90%) with CoronaVac™ and Pfizer-BioNTech™.

A significant difference was observed in the age distribution when comparing the groups (p<0.001). As presented in Table 1, the median ages, along with their respective ranges, for the NoVac, PV, and FV groups were 51 (18-98), 61 (21-91), and 71 (23-99) years, respectively. Furthermore, when comparing the time interval between the date of the last vaccination and the date of hospital admission, a significant difference emerged between the FV and PV patients. The respective durations for FV and PV patients were 124 (14-278) days and 35 (14-259) days (p<0.001; Table 1).

**Table 1:** Comparison of the data between the vaccination groups SD: standard deviation; ICU: intensive care unit; WBC: white blood cells; BUN: blood urea nitrogen; ALT: alanine transaminase; AST: aspartate transaminase; ESR: erythrocyte sedimentation rate; CRP: C-reactive protein; IL-6: interleukin 6; P1: probability among all groups; P2: probability of unvaccinated vs. fully vaccinated; P3: probability of unvaccinated vs. partially vaccinated; P4: probability of fully vaccinated vs. partially vaccinated

	Unvaccinated (n=1321)		Partially vaccinated (n=214)		Fully vaccinated (n=944)		Total (n=2479)		P1	P2	P3	P4
	Mean±SD	Median (min-max)	Mean±SD	Median (min-max)	Mean±SD	Median (min-max)	Mean±SD	Median (min-max)				
Age (in years)	51.65±16.57	51 (18-98)	55.76±13.99	61 (21-91)	68.49±12.62	71 (23-99)	58.42±16.95	59 (18-99)	0.000	0.000	0.000	0.000
Days from the last vaccination	N/A	N/A	48.85±41.74	35 (14-259)	131.26±63.05	124 (14-278)	116.03±67.72	108.5 (14-278)	0.000	N/A	N/A	0.000
Inpatient duration	7.51±5.72	6 (0-67)	6.48±4.78	5 (0-36)	8.13±6.14	4 (0-55)	7.66±5.83	7 (0-67)	0.000	0.007	0.010	0.000
ICU duration	2.75±6.48	0 (0-67)	2.88±8	5 (0-67)	3.58±7.98	8 (0-67)	3.08±7.23	0 (0-67)	0.016	0.005	0.938	0.136
Inpatient+ICU	10.25±7.83	8 (1-71)	9.36±8.63	13 (1-67)	11.69±9.15	14 (1-78)	10.72±8.46	8 (1-78)	0.000	0.000	0.002	0.000
WBC (thousand/mm^3^)	7.45±4.67	6.43 (0.55-92.52)	8.06±5	7.97 (1.27-47.09)	9.32±8.04	8.18 (0.27-119.31)	8.21±6.26	7.07 (0.27-119.31)	0.000	0.000	0.051	0.001
Neutrophil (thousand/mm^3^)	5.71±4.36	4.69 (0.07-86.09)	6.12±4.01	6.6 (0.24-23.1)	6.97±4.7	6.42 (0.01-44.54)	6.23±4.5	5.21 (0.01-86.09)	0.000	0.000	0.068	0.003
Lymphocyte (thousand/mm^3^)	1.22±0.88	1.1 (0.08-24.04)	1.37±2.76	0.71 (0.15-40.1)	1.67±5.95	0.87 (0.04-110.24)	1.4±3.82	1.1 (0.04-110.24)	0.195	0.290	0.202	0.103
Monocyte (thousand/mm^3^)	0.48±0.33	0.39 (0.03-3.3)	0.51±0.35	0.39 (0.05-2.38)	0.6±0.73	0.46 (0.01-19.37)	0.52±0.52	0.44 (0.01-19.37)	0.000	0.000	0.185	0.001
Hemoglobin	13.09±2.03	13.2 (4.7-19.1)	13.21±1.99	12.6 (7.3-17.5)	12.41±2.08	12.2 (4.6-18.5)	12.84±2.07	13 (4.6-19.1)	0.000	0.000	0.614	0.000
Platelet (thousand/mm^3^)	232.05±101.1	212 (24-824)	233.46±118.49	208 (5-801)	238.77±118.1	196 (1-1365)	234.73±109.41	213 (1-1365)	0.654	0.443	0.740	0.470
BUN	16.91±14.46	12.9 (2.4-135.7)	19.49±17.81	16.4 (2.8-144)	24.88±17.49	23.9 (3.8-147.8)	20.17±16.41	15.2 (2.4-147.8)	0.000	0.000	0.001	0.000
Creatinine (mg/dL)	1.01±0.87	0.85 (0.16-10.58)	1.09±0.81	0.97 (0.19-6.01)	1.34±1.17	1.15 (0.3-11.08)	1.14±1	0.92 (0.16-11.08)	0.000	0.000	0.015	0.000
ALT	46.76±136.67	28 (5-3885)	45.95±87.71	22 (5-902)	31.51±49.29	21 (5-888)	40.89±107.68	25 (5-3885)	0.000	0.000	0.260	0.000
AST	56.84±192.97	35 (6-4708)	54.81±161.15	30 (6-1710)	42.68±91.99	32 (6-1892)	51.28±159.2	32 (6-4708)	0.000	0.000	0.001	0.472
ESR (mm/h)	48.04±25.07	45 (2-140)	47.59±25.75	45 (2-126)	52.89±27.37	55 (2-140)	49.85±26.12	47 (2-140)	0.000	0.000	0.910	0.022
CRP	79.59±67.53	61.5 (0-450)	92.38±81.64	83.3 (0.6-400)	109.16±86.52	101.5 (0-409.9)	91.94±77.74	70.9 (0-450)	0.000	0.000	0.161	0.004
Procalcitonin	0.56±3.73	0.07 (0.02-96.1)	1.08±5.96	0.21 (0.02-74)	1.05±5.79	0.32 (0.02-100)	0.79±4.83	0.09 (0.02-100)	0.000	0.000	0.042	0.010
Ferritin	678.85±981.84	421 (6-19436)	1189.69±6919.28	480 (17-100000)	676.15±1406.75	441.4 (6-18675)	722.13±2328.45	395 (6-100000)	0.008	0.002	0.810	0.114
IL-6	308.33±693.54	40.8 (1.5-5000)	298.45±843.1	36.2 (1.8-5000)	333.55±754.97	70.4 (1.7-5000)	317.05±730.78	47.25 (1.5-5000)	0.042	0.029	0.456	0.060
D-dimer	1.2±2.18	0.53 (0.18-20.3)	1.61±2.85	1.21 (0.2-20.3)	1.52±2.47	0.91 (0.2-20.9)	1.36±2.36	0.59 (0.18-20.9)	0.000	0.000	0.020	0.417
Fibrinogen	542.65±167.78	535 (100-900)	555.37±189.6	570 (184-900)	580.76±173.54	578 (100-900)	558.6±172.81	550 (100-900)	0.016	0.004	0.518	0.339
Troponin T	23.79±101.68	6.7 (3-1895)	33.16±95.55	12.4 (3-759)	48.39±218.61	24.3 (3-4723)	33.96±156.85	10.2 (3-4723)	0.000	0.000	0.001	0.000

There was a significant difference noted in the length of stay in both the inpatient clinic and the ICU (p<0.001 and p=0.016, respectively). However, when comparing the duration of ICU hospitalization, no significant difference was observed among the PV, FV, and NoVac patient groups (Table 1).

For the complete blood count (CBC) parameters, which include white blood cells (WBC), neutrophils, lymphocytes, monocytes, and platelets (Plt), a significant difference was observed among the groups in all parameters except for lymphocyte counts (p<0.05; Table 1). The intergroup comparisons are detailed in Table 1. Furthermore, significant differences were noted in kidney parameters (blood urea nitrogen (BUN), creatinine) and liver parameters (aspartate transaminase (AST), alanine transaminase (ALT)) across the groups (p<0.05; Table 1).

Inflammatory markers, including erythrocyte sedimentation rate (ESR), C-reactive protein (CRP), procalcitonin, ferritin, and IL-6, as well as coagulation parameters like D-dimer and fibrinogen and cardiac marker troponin T were observed to be higher in FV patients compared to NoVac and PV patients. Notably, ferritin and D-dimer levels were exceptions, being higher in PV patients (p<0.05; Table 1).

The rate of infection with the delta variant of COVID-19 was observed to be higher in FV patients (76.6%) compared to NoVac (58.4%) and PV (50.9%) patients (p<0.05; Table 2).

**Table 2:** Comparison of demographic and outcome data among the groups ICU: intensive care unit

		Unvaccinated n(%)	Fully vaccinated n(%)	Partially vaccinated n(%)	Total n(%)	p
Gender	Male	683 (51.7)	481 (51)	114 (53.3)	1278 (51.6)	0.818
	Female	638 (48.3)	463 (49)	100 (46.7)	1201 (48.4)	
Age>65	<65	1028 (77.8)	290 (30.7)	169 (79)	1487 (60)	<0.001
	≥65	293 (22.2)	654 (69.3)	45 (21)	992 (40)	
ICU requirement	No	1013 (76.7)	674 (71.4)	162 (75.7)	1849 (74.6)	0.016
	Required	308 (23.3)	270 (28.6)	52 (24.3)	630 (25.4)	
Outcome	Exitus	180 (13.6)	220 (23.3)	37 (17.3)	437 (17.6)	<0.001
	Discharged	1141 (86.4)	724 (76.7)	177 (82.7)	2042 (82.4)	
Delta variance	No delta	550 (41.6)	222 (23.5)	105 (49.1)	877 (35.4)	<0.001
	Delta	771 (58.4)	722 (76.5)	109 (50.9)	1602 (64.6)	
Blood type	0 Rh(+)	143 (10.8)	125 (13.3)	26 (12.1)	294 (11.9)	0.961
	A Rh(+)	212 (16)	185 (19.6)	41 (19.2)	438 (17.7)	0.702
	B Rh(+)	76 (5.8)	71 (7.5)	12 (5.6)	159 (6.4)	0.847
	AB Rh(+)	39 (3)	36 (3.8)	5 (2.3)	80 (3.2)	0.741
	0 Rh(-)	15 (1.1)	16 (1.7)	2 (0.9)	33 (1.3)	0.765
	A Rh(-)	39 (3)	27 (2.9)	5 (2.3)	71 (2.9)	0.522
	B Rh(-)	10 (0.8)	14 (1.5)	3 (1.4)	27 (1.1)	0.482
	AB Rh(-)	6 (0.5)	5 (0.5)	1 (0.5)	12 (0.5)	0.994

No relationship was observed between vaccination status and blood type (Table 2). The mortality rate according to COVID-19 infection was higher in FV (23.3%) patients compared to PV (17.3%) and NoVac (13.6%) patients (p<0.05; Table 2).

## Discussion

In the FV group, patients tended to be older, exhibited a higher need for intensive care, and had longer clinical and ICU hospital stays. Additionally, the mortality rate in the FV group surpassed that of the other groups. Acute-phase reactants, as well as BUN and creatinine values measured upon admission, were also notably elevated in the FV group.

This study does not aim to compare the protective effect of vaccination status against COVID-19 infection. Notably, vaccine efficacy studies have been conducted across both young and elderly populations. These studies have demonstrated that vaccination is protective across all age groups; in fact, the unvaccinated population was found to be hospitalized 12 times more frequently than those who were vaccinated [16-19]. Our study provides insight into the general characteristics of COVID-19 patients who were hospitalized over a seven-month period. Additionally, it allows for a detailed examination of the laboratory values and clinical progression of those with breakthrough infections. By understanding this data, clinicians can gain guidance on several fronts, including the potential need for booster doses, enhanced assessments in emergency rooms, meticulous monitoring of laboratory results, closer scrutiny for ICU requirements, and timely initiation of anti-cytokine or steroid treatments for the most vulnerable populations.

The median age of our FV group was 71 years, in line with previous publications [16,19-22]. An analysis showed that more than two-thirds of FV patients hospitalized with COVID-19 infection were patients over the age of 65 [20,21]. In our study, 69.3% of the FV COVID-19 patients were above the age of 65. Existing studies have highlighted that the mortality rate escalates with advancing age, establishing age as an independent risk factor for COVID-19-related mortality [19]. The observed mortality rate for hospitalized patients with breakthrough infections aged between 65 and 80 was 17.7%. Notably, this rate escalates to 30.6% for patients who are aged over 80 years [23].

A decline in innate immunity coupled with a diminished adaptive immune response might account for the elevated incidence of breakthrough infections observed in individuals of advanced age. In comparison to younger individuals, elderly patients exhibit significantly reduced levels of serum IgG, peripheral IgG, IgM, CD19+ memory B cells, and IL-2-producing CD4+ T cells post vaccination. This results in diminished antibody levels and antibody affinity [24]. The efficacy of the mRNA vaccine in averting the disease within the geriatric population has been observed to decline to 79.8% [25], and its efficacy in preventing hospitalization among patients aged over 75 was further diminished, standing at 76% [26]. Our study indicates that full vaccination offers protection against hospitalization for patients below the median age of 71 years. However, the elevated hospitalization rate observed in older patients, despite being FV, suggests an inability to generate adequate antibodies in response to the vaccine, making them more susceptible to the disease.

It has been shown that anti-spike antibody levels were observed as declined of more than half within 70 days post vaccination [27], and the effectiveness may reduce significantly after about four months [27,28]. A direct relationship was found between the time elapsed after vaccination and infection and severe illness [27-30]. In our study, the median time interval between the date of the last vaccination and the onset of infection within the FV group was 124 days. Suleyman et al. [19] reported this duration as 135 days and emphasized the longer post-vaccination duration in non-survived COVID-19 patients. Given this data, the diminishing concentration of antibodies over time might be a contributing factor to the severity of COVID-19 infections necessitating hospitalization, as well as the elevated mortality rates observed among our FV patients.

A study conducted in China showed that neutralizing antibody levels, which decreased after six months in individuals over 60 years of age, increased rapidly following the administration of a booster dose [31]. Another research study highlighted that the administration of a booster vaccine provided an 11-fold increase in protection against symptomatic COVID-19 infection and a 19-fold enhancement in protection against severe manifestations of the disease [32]. Initially, at the start of our study in May 2021, only two doses were administered as part of the routine vaccination process. However, by July 2021, the Ministry of Health of Turkey introduced a third dose to the vaccination regimen [11].

Inactivated vaccines, which have received emergency use authorization, are the most widely administered vaccines globally. In Turkey, the vaccination program initiated in January 2021 began with the use of CoronaVac™, and, as a result, this vaccine was administered to a larger portion of the population [7,11]. Consequently, the majority of patients in our study, specifically 87.30%, received the CoronaVac™ vaccine. Research conducted in Brazil revealed that the predominant group among patients hospitalized with breakthrough infections had received inactive vaccines. Furthermore, the efficacy of inactive vaccines in preventing hospitalization, severe illness, and death was found to be lower compared to other vaccine types [33]. In a separate research study, the efficacy of CoronaVac™ was determined to be 46.8% for individuals aged over 70 years [34]. Additionally, it was reported that the vaccine's efficacy notably diminished with increasing age [34]. Research has demonstrated that the most efficacious vaccine against COVID-19 infection is the mRNA vaccine and it is followed in effectiveness by the adenovirus vaccine and subsequently by the inactivated vaccines [35]. One interpretation of the data suggests that while vaccination initially demonstrated sufficient efficacy against early virus strains, it may not have elicited an adequate immune response to counter subsequent emerging strains. In our study, a significant portion of the FV group (87.30%) were vaccinated with CoronaVac™, and most of the patients in this group were tested positive for the delta variant (76.6%). This situation can be interpreted as an indication that the vaccine offers diminished protection against this newer strain, leading to more severe disease progression and higher mortality rates.

Throughout the study period, spanning from May 1, 2021, to December 1, 2021, the delta variant gained has become dominant not only in Turkey but also globally. Concurrent with the propagation of the third wave, our hospital experienced a noticeable surge in hospitalizations, commencing in August 2021. The delta variant was detected in 64% of the patients included in the study. Specifically, the rate of delta variant positivity was 76.6% in the FV group and 58.4% in the PV group. Many studies have reported decreased efficacy of vaccines against the delta variant and high infectivity of the variant [36,37]. It has been documented that the efficacy of the CoronaVac™ against severe delta variant infection, particularly in elderly individuals, is 79% within the first month; however, this efficacy gradually diminishes over time to reach 24% [38]. The notably high delta positivity rate observed in the FV group indicates a reduced efficacy of the vaccine against it. Factors such as advanced age, the potential limited effectiveness of CoronaVac™ against the delta variant, and the duration since the last vaccine dose may contribute to elucidating the unfavorable clinical progression and elevated mortality rate observed within the FV patient group.

In FV patients, in accordance with existing literature, troponin T, fibrinogen, acute-phase reactants (including ESR, CRP, and procalcitonin), IL-6, BUN, creatinine, and WBC values exhibited higher levels, while hemoglobin (Hgb) and Plt values were lower compared to the other groups [21,39,40]. During the clinical follow-up of FV patients, despite the shorter inpatient clinic stay (four days), the need for ICU (28.6%), ICU hospitalization times (eight days), total hospital stay (14 days), and mortality rates (23.3%) were significantly higher than the other groups. This observation suggests that these patients presented at the emergency department with a more severe clinical condition, as indicated by higher acute-phase reactants at admission. The FV patient also displayed rapid progression during their hospital stays, characterized by an average hospitalization duration of four days, and a considerable proportion required admission to the ICU. Likewise, a study conducted by Bahl et al. [13], encompassing an analysis of 11,834 patients, revealed that the FV group exhibited a significantly lower admission and hospitalization rate in cases of COVID-19 infection when compared to the unvaccinated UV group, with a reduction of 96%. However, the FV patients admitted to hospitals displayed higher requirements for ICU care and experienced a higher mortality rate than individuals in the other groups.

Our study have several limitations. It is important to note that this study is single-centered and retrospective in nature. Additionally, due to the pandemic conditions, it was not feasible to acquire patient demographic data such as weight, height, body mass index, as well as information regarding smoking, alcohol consumption, medication history, and comorbid conditions from the EMR.

## Conclusions

As per the findings of the current study, complete vaccination significantly reduces hospitalization rates for individuals below the median age of 71 years. Conversely, patients of advanced age exhibited higher rates of hospitalization, an increased need for intensive care, prolonged hospital stays, elevated inflammatory markers, and increased mortality rates, even in cases of full vaccination. Consequently, it is imperative to conduct a comprehensive and meticulous assessment of elderly patients in the emergency department to mitigate morbidity and mortality and initiate treatment promptly. It's worth noting that, as of now, there is insufficient evidence to recommend the discontinuation of primary prevention measures, such as social distancing, mask usage, and other protective interventions, in addition to considering booster vaccination doses for elderly patients.
